# Prevalence and associated factors of pterygium among adults living in Gondar city, Northwest Ethiopia

**DOI:** 10.1371/journal.pone.0174450

**Published:** 2017-03-30

**Authors:** Dereje Hayilu Anbesse, Tsehay Kassa, Biruktayit Kefyalew, Atirsaw Tasew, Abie Atnie, Beredu Desta

**Affiliations:** Department of Optometry, School of Medicine, College of Medicine Health Science, University of Gondar, Gondar city, Ethiopia; Medizinische Universitat Graz, AUSTRIA

## Abstract

**Purpose:**

The aim of this study was to assess the prevalence and associated factors of pterygium among adults living in Gondar city, Northwest Ethiopia.

**Methods:**

A cross sectional design study was carried out in 390 participants in Gondar city from April 15 to May 7, 2016. Basic ophthalmic examination was performed using portable slit lamb, 3.5x magnifying loop with torch light and a pretested and structured questionnaire was completed. The raw data has been entered into EPI INFO 3.5.1 and analyzed by SPPSS version 20. Descriptive statistics was summarized descriptive data. Logistic regression was used to summarize the predictors of pterygium. The variables with p-value less than 0.05 were considered as significant risks of pterygium.

**Result:**

The prevalence of pterygium among study participants was 151(38.7% (95%CI; 33.8–43.8)). Among those who have pterygium, 149(98.7%) were developed pterygium on the nasal side and 15(9.9%) on temporal side of the either eye and 13(8.6%) have both. Age between 41-60(AOR = 2.20(95%CI: 1.22, 3.39)), age between 61-86(AOR = 7.97(95%CI: 2.74, 23.17)), male sex (AOR = 2.20(95%CI: 1.28, 3.82)), outdoor working area(AOR = 3.75(95%CI: 2.18, 6.46)), the use of traditional eye medication (AOR = 2.55 (95%CI: 1.04, 5.90)) and family history of pterygium (AOR = 6.68(95% CI: 2.53, 17.60)) were positively associated with pterygium whereas use of sunglass/hat (AOR = 0.40(95%CI:0.20, 0.78)) was negatively associated.

**Conclusion:**

There is a high prevalence of pterygium in Gondar city northwest Ethiopia. Old age, male sex, outdoor working area, utilization of traditional eye medication and family history of pterygium were statistically significant predictors of pterygium. The use of sunglass/hat was protective against pterygium.

## Introduction

Pterygium is a wingy shaped fibro vascular growth of the conjunctiva on to the cornea usually nasally. It occurs in the palpebral fissure area, much more often nasally than temporally, although either or both (“double” pterygium) can occur [1]. It can induce significant astigmatism and cause visual impairment. Pterygium is often preceded by a related non-cancerous condition called pingueculum. Its main clinical presentations are redness, irritation, decreased vision and ocular discomfort. It may also be asymptomatic [2, 3].

If pterygium is left untreated and its associated risk factors are not avoided or reduced, it can result in visual impairment or blindness due to fibro vascular coverage of conjunctiva over the visual axis of the cornea. This is due to the induction of astigmatism and opacity [4, 5]. It is a common external ocular disease with prevalence ranging between 0.3% and 36.6% globally. The prevalence rate of pterygium varies widely with the variation of altitude, age, gender, occupation and socio economic status. The prevalence rate of 3.0% among patients attending ophthalmology clinic in Ankara, Turkey, 12.5% among motorcyclists in Benin city, Nigeria and 8.8% in Meskan district of Southern Ethiopia were reported [6–8].

Even though the aetiology of pterygium is ill defined, there are factors which associated with formation of pterygium such as outdoor working environment, age being old, male sex, living in an area with higher exposure of ultraviolet radiation, dry and windy climate [9–11]. The study conducted in Blue Mountain, Australia, found a significant association between pterygium and increased pigmentation (skin and hair color), decreased skin sun sensitivity and sun related skin damage [12]. The study area is located in the tropics, where the prevalence of pterygium is not well studied previously especially the associated factors of pterygium. Therefore, this study aimed to determine the magnitude of pterygium and its associated factors among adults so that awareness creating about avoidance of risk factors, regular screening programs for prevention and early intervention will be planned and implemented.

## Materials and methods

### Study design, setting and sampling

A population based cross sectional design study was conducted with adults in Gondar city from April 15 to May 7, 2016. Gondar city is located in North Gondar zone 748 km from the capital city Addis Ababa with a total population of 225,125. It has an altitude of 2,200m above sea level with warm and dry weather condition. It has 10 sub-cities hosting approximately 53,725 households. There is one tertiary eye care and training center with a catchment population of about 14 million.

A total of 425 sample size was determined by single proportional formula by considering 10% non response rate and two design effect. In the study, 399 study participants were recruited and completed a questionnaire along with a basic ophthalmic examination. This corresponds to a 91.76% response rate. A Multistage systematic random sampling technique using two stages of sampling process has beenused. First, four kebeles (smallest administrative unit hosting about 2000 households) out of twenty four kebeles was selected using simple random sampling method. Then systematic random sampling method was used to select participating households proportionally (every 24^th^ households). Finally one adult in each participating household with age greater than 20 years old was randomly selected and recruited as study participant.

The study was conducted in accordance with the Declaration of Helsinki and approved by the University of Gondar Ethical Review Board. In accordance with the Ethiopian National Research Ethics Review Guideline, verbal informed consent was obtained from all adults greater than age 20 years old using an information sheet in the local language “Amharic”. Since the study didn’t involve invasive eye examination procedures, the university ethical review board approved verbal informed consent. The study participants’ agreements were first obtained verbally prior to data collection. Then the data was collected by trained senior optometrists. Those adults who had pterygium were prescribed sunglass and referred to the University of Gondar tertiary eye care and training center for detail examination and management.

### Definition of pterygium

Pterygium: defined as presence of any size of wingy shaped fibro-vascular growth of the conjunctiva that extends to the cornea and/ or those individuals who have history of pterygium surgery

Grade one: wingy shaped fibro-vascular growth of the conjunctivaextends less than 2 mm onto the cornea

Grade two: wingy shaped fibro-vascular growth of the conjunctivathat involves up to 4 mm of the cornea

Grade three: wingy shaped fibro-vascular growth of the conjunctiva that encroaches onto more than 4 mm of the cornea and involves the visual axis

### Data collection

The pre-tested and structured questionnaire of local language ‘Amharic’ was used to carry out interview with adults greater than 20 years old (S1 Questionnaire). Regular check-up for completeness and consistency of the data was made on daily basis. On the field work, data quality was insured through cross-checking 5% of the sample by principal investigators. The collected data has been checked for accuracy and completeness by the principal investigators. The data collected from the study participants included: socio economic and demographic factors, behavioral and environmental factors. Standard basic ophthalmic examinations by using portable slit lamb, 3.5X magnified loop, and torch light were done for all participants by senior Optometrist. Examination findings were recorded in English.

### Statistical analysis

The raw data has been entered into EPI INFO 3.5.1. After data was coded and cleaned, it was exported to and analyzed by using SPSS version 20. Descriptive factors have beensummarized by frequency and proportions, and summary statistics such as mean, standard deviation and ranges. The analytical statistics was done by using bivariate and multivariate logistic regression. Those variables with 95% CI and p-value less than 0.05 were considered as statistically significant factors of pterygium.

## Results

A total of 390 study participants with response rate of 91.76% were involved in the study. Among them 222 (56.9%) of the respondents were females. The mean age of study participants was 38.69 ± 15.83 (range 20 to 88) years. The majority of respondents 120 (30.8%) were illiterate and 123 (31.5%) of them were merchants in occupation. Majority of the respondents 295 (75.6%) were orthodox and 83 (21.3%) of the respondents were Muslims. Table 1.

**Table 1:** Socio demographic characteristics of study participants among adults living in Gondar city, Northwest Ethiopia, 2016.

Variables	Frequency	Percentage
Age		
20–40	249	63.8
41–60	105	26.9
61–88	36	9.2
Sex		
Male	168	43.1
Female	222	56.9
Marital status		
Single	112	28.7
Married	209	53.6
Divorced	31	7.9
Widowed	38	9.7
Educational status		
No	120	30.8
Religious education	20	5.1
Primary school	110	28.2
Secondary school	96	24.6
College/university	44	11.3

The prevalence of pterygium among study participants was 151(38.7% (95%CI; 33.8–43.8)). Among those who have pterygium, 149(98.7%) were developed pterygium on the nasal side and 15(9.9%) on temporal side of the either eye and 13(8.6%) have both. One fourth of them 97 (24.87%) had grade one. Table 2.

**Table 2:** Magnitude, location and grades of pterygium among study participants of adults living in Gondar city, Northwest Ethiopia, 2016.

Variables	Frequency	Percent
Pterygium(n = 390)		
Yes	151	38.7
No	239	61.3
Pterygium location		
Nasal	149	98.7
Temporal	15	9.9
Pterygium grade		
Grade one	97	64.2
Grade two	52	34.4
Grade three	9	5.9

In bivariate analysis, old age, sex being male, marital status being single, divorced and widowed, educational status being no, primary and religious, outdoor working area, large family size, medium monthly income, current drinking alcohol, past and current smoking, family history of pterygium, the use of traditional medication were associated with pterygium. In multivariate logistic regression; old age, sex being male, outdoor working area, the use of sunglass/hat, the use of traditional eye medication and family history of pterygium were statistically and independently associated with pterygium.

As a result, those participants with early old age (41–60 years) are 2.20 times more likely to develop pterygium as compared to those adults (20–40) years of age (AOR = 2.20(95%CI: 1.22, 3.39)). Those with late old age (61–86 years)are 7.97 times more likely to develop pterygium as compared to those adults years of age (AOR = 7.97(95%CI: 2.74, 23.17)). Male sex is 2.20 times more likely to have pterygium than females (AOR = 2.20(95%CI: 1.28, 3.82)). Outdoor working environment is 3.75 more likely to have pterygium as compared to indoor working area (AOR = 3.75(95%CI: 2.18, 6.46)). In this study, the use of sunglass/hat is 0.40 less likely to have pterygium as compared to non-using (AOR = 0.40(95%CI: 0.20, 0.78)). The use of traditional eye medication is 2.55 times more likely to develop pterygium than non-user (AOR = 2.55 (95%CI: 1.04, 5.90)). Positive family history is 6.68 more likely to have pterygium than negative family history (AOR = 6.68(95% CI: 2.53, 17.60)). Table 3.

**Table 3:** Factors associated with pterygium of study participants among adults living in Gondar city, Northwest Ethiopia, 2016.

Variables	Pterygium				
	Yes	No	COR(95%CI)	AOR (95%CI)	p-value
Age					
20–40	69	180	1.00	1.00	
41–60	54	51	2.76(1.72, 4.43)	2.20(1.22, 3.39)	0.009
61–86	28	8	9.13(3.97, 21.00)	7.97(2.74, 23.17)	0.000
Sex					
Male	85	83	2.42(1.59, 3.67)	2.20(1.28, 3.82)	0.005
Female	66	156	1.00	1.00	
Marital status					
Single	18	94	1.00		
Married	95	114	4.35(2.45, 7.72)		
Divorced	17	14	6.34(2.66, 15.12)		
Widowed	21	17	6.45(2.86, 14.56)		
Educational status					
No	58	62	4.21(1.80, 9.80)		
Religious	13	7	8.36(2.53, 27.64)		
Primary	45	65	3.11(1.32, 7.33)		
Secondary	27	69	1.76(0.73, 4.27)		
College/university	8	36	1.00		
Family size					
0–3	61	127	1.00		
4–6	65	98	1.38(0.89, 2.14)		
7–10	25	14	3.72(1.80, 7.65)		
Monthly income					
30–1000	107	170	0.63(0.21, 1.84)		
1001–3000	37	62	0.60(1.94, 1.84)		
3001–6000	7	7	1.00		
Working area					
Indoor	60	166	1.00	1.00	
Outdoor	91	73	3.45(2.25, 5.28)	3.75(2.18, 6.46)	0.000
Dust exposure					
no	61	116	1.00		
Yes	90	123	1.39(0.92, 2.10)		
Use of sunglass/hat					
No	125	186	1.00	1.00	
Yes	26	53	0.73(0.43, 1.23)	0.40(0.20, 0.78)	0.007
Smoking					
Never	131	229	1.00		
Past	10	5	3.50(1.17, 10.45)		
Current	10	5	3.50(1.17, 10.45)		
Alcohol drinking					
Never	99	188	1.00		
Past	21	21	1.90(0.99, 3.64)		
Current	31	30	1.96(1.12, 3.43)		
Use of traditional eye medication					
No	126	227	1.00	1.00	
Yes	25	12	3.75(1.82, 7.73)	2.55(1.04, 5.90)	0.028
Family history of pterygium					
No	124	231	1.00	1.00	
Yes	27	8	6.29(2.77, 14.26)	6.68(2.53, 17.60)	0.000

## Discussion

The prevalence of pterygium among study participants was 151(38.7% (95%CI; 33.8–43.8)). This is one of the highest magnitudes of pterygium among different epidemiological studies. It is in line with the study conducted in Amazon forest of Brazil (36.6%) and in the rural area of Doumen county, China (37.46%) [13,14]. In comparison to the study conducted in Japanese population aged 40 years and above (30.8%), the present day result is higher [9]. There were many epidemiological studies showing low prevalence of pterygium. For instance the prevalence rate of pterygium was 19.6% in central Myanmar, 12.5% in Nigeria, 8.8% southern Ethiopia and 8.47% in central India [5, 7, 8, 10]. The discrepancy observed might be due to the variation of geographical and climatic setting, sun light and ultraviolet exposure, age, economic situation and the use of traditional medication.

Those participants with early old age (41–60 years) were 2.20 times more likely to develop pterygium as compared to adults (20–40) years of age (AOR = 2.20(95%CI: 1.22, 3.39)). Those with late old age (61–86 years) were 7.97 times more likely to develop pterygium as compared to those adults years of age (AOR = 7.97(95%CI: 2.74, 23.17)). There were a plenty of previous studies which support this finding. The south western Island of Japan and Barbados Eye Study which published in 2009 and 2001 respectively reported a positive association between pterygium and an old age [9, 15]. As age increases the exposure to predictors such as ultraviolet light, outdoor working habit, dust particles will increase the precipitation of pterygium.

Male sex was 2.20 times more likely to have pterygium than females (AOR = 2.20(95%CI: 1.28, 3.82)). The present finding was correlated with the Southern Harbin eye study and Beijing eye study which indicated that men population were heavily engaged in outdoor work activities and exposed to dust particles and ultraviolet light so that they are more prone to be affected by pterygium. On the contrary, the two studies done in China reported that women are at higher risk than men to develop pterygium. This is due to that in Tibet, women are fully engaged in outdoor job and traditionally they do not wear sunglass [16, 17].

The recent study found positive association between outdoor working environment and pterygium (AOR = 3.75(95%CI: 2.18, 6.46)). Different authors reported similar finding. At an outdoor environment, high light reflectivity, including from sand and water cause damage to limbal stem cells by ultraviolet light and by activation of matrix metalloproteinase and leads to pterygium [18].

The other result from recent study was, the negative association between the use of sunglass/hat and pterygium (AOR = 0.40(95%CI: 0.20, 0.78)). This result is conjugate with the reports of Barbados eye study and study among motorcyclist in Nigeria and together they suggested that the use of sunglass/hat reduces the risk of developing pterygium by absorbing and reducing the exposure of harmful ultraviolet light [7, 19].

The utilization of traditional eye medication was also one of the positive predictors of pterygium in this study (AOR = 2.55 (95%CI: 1.04, 5.90)). The previous study done in Limpopo province of South Africa reported the similar result which suggested that the use of traditional eye medicine implicates hereditary predisposition to pterygium occurrence [20].

Finally, positive family history was positively associated with pterygium development (AOR = 6.68(95% CI: 2.53, 17.60)). The Limpopo study also reported the same finding. This might be due to that pterygium is hereditary and hared environment of the affected individuals and their family [20].

The current study has some important limitations: Some of the data were self-reported and subject to recall bias from participants. We didn’t conduct laboratory investigations to explore other related data with pterygium.

## Conclusion

There is a high prevalence of pterygium in Gondar city northwest Ethiopia. Old age, male sex, outdoor working area, utilization of traditional eye medication and family history of pterygium were statistically significant predictors of pterygium. The use of sunglass/hat was a significant protective factor for pterygium occurrence.

## Supporting information

S1 Questionnaire Questionnaire and data extraction form to study prevalence and associated factors of pterygium at Gondar city, Northwest Ethiopia, 2016. (DOCX)
