# Supplementary material for: Prevalence and associated factors of pterygium among adults living in Gondar city, Northwest Ethiopia
Source: PLoS One. 2017 Mar 30;12(3):e0174450. doi: 10.1371/journal.pone.0174450 (PMC5373563; doi:10.1371/journal.pone.0174450)
Supplement: S1 Questionnaire — (DOCX) [file pone.0174450.s001.docx]

**Questionnaire and data extraction form to study prevalence and associated factors of pterygium at Gondar city, Northwest Ethiopia, 2016**

Good morning/Afternoon, our name is -----------------, we are optometrist and research staffs of University of Gondar Collage of medicine and health sciences. We interested in learning more about prevalence and associated factors of pterygium. The data will be collected by using questionnaire and data extraction format which was designed for a research work and approved by University of Gondar College of Medicine and health Sciences ethical review board. The information you provide us is completely confidential and will not be shared with anyone else without your consent. Your name or any identifying information will not be registered. You may refuse to answer any question and choose to stop the interview at any time. The information you provide us is extremely important and valuable, as it will help the concerned body. You have full right to refuse, to take part or to interrupt the interview at any time. We hope you will help us by completing this survey. We thank you in advance for taking your time to participate in providing data. Next, we will read a consent, which assures your interest to participate.

Do I have your permission to continue?

If yes, thank you and continue. If no, thank you and go to next study subject.

Data collector

Name ----------------------- signature ------------------------ date -------------------

Checked by supervisor

Name --------------------- signature---------------------- date-------------------

1. **Socio demography**
2. Age in years -------------------
3. Sex

- Male
- Female

1. Address

- Rural
- Urban

1. Marital status

- Single
- Married
- Widowed
- Divorced

1. Educational status

- No formal education
- Primary school
- Secondary school
- College/university

1. Religion

- Orthodox
- Muslim
- Catholic
- Protestant
- Other

1. Occupation

- Student
- Teacher
- Farmer
- Merchant
- Employed
- Housewife
- No
- Other

1. Monthly income in ETB---------------
2. **Environmental characteristics**
3. Working area

- Outdoor
- Indoor

1. Dust exposure

- Yes
- No

1. **Behavioral characteristics**
2. The use of eye glass/hat

- Yes
- No

1. Drinking alcohol

- Never
- Past
- Current

1. Smoking

- Never
- Past
- Current

1. The use of any traditional eye medication

- Yes
- No

1. Symptoms for dry eye syndrome

- Feeling of Dryness
- Burning and grittiness
- Redness
- Tearing

1. Family history of Pterygium (among first degree relatives) please show them the picture

- Yes
- No

1. **Form for clinical features for pterygium study in Gondar city, 2016**

|  | ***Symptoms related to pterygium*** | RE | LE | Remark | |
| --- | --- | --- | --- | --- | --- |
|  | Redness |  |  |  | |
|  | Feeling of dryness |  |  |  | |
|  | Vision reduction |  |  |  | |
|  | Irritation |  |  |  | |
|  | Grittiness |  |  |  | |
|  | ***Anterior segment examination*** |  |  |  | |
|  | ***Bulbar conjunctiva*** |  |  |  | |
|  | Redness |  |  |  | |
|  | Radial vascularization of fleshy growth |  |  |  | |
|  | Wedge shaped fleshy translucent membrane with apex extending towards the cornea |  |  |  | |
|  | Others |  |  |  | |
|  | Normal |  |  |  | |
|  | ***Limbus*** |  |  |  | |
|  | Obscured by vascularized fleshy growth |  |  |  | |
|  | Others |  |  |  | |
|  | Normal |  |  |  | |
|  | ***Cornea*** |  |  |  | |
|  | presence of any size of wingy shaped fibro-vascular growth of the conjunctiva that extends to the cornea with its apex |  |  |  | |
|  | Covered by wingy shaped fibro-vascular growth of the conjunctiva extends from limbus up to 2 mm onto the cornea |  |  |  | |
|  | Covered by wingy shaped fibro vascular growth of the conjunctiva that involves up to 4 mm of the cornea |  |  |  | |
|  | Covered by wingy shaped fibro-vascular growth of the conjunctiva that encroaches onto more than 4 mm of the cornea and involves the visual axis |  |  |  |  |
|  | Others |  |  |  |  |
|  | Normal |  |  |  |  |
|  | Final assessment |  |  |  |  |
|  | Pterygium |  |  |  |  |
|  | Other eye disease |  |  |  |  |
|  | Normal |  |  |  |  |
|  | If pterygium , specify the grade |  |  |  |  |
|  | Grade 1 |  |  |  |  |
|  | Grade 2 |  |  |  | |
|  | Grade 3 |  |  |  | |

## Mark ‘X’ for the selected option in the table
